# An Exploration of Social Functioning in Young People with Eating Disorders: A Qualitative Study

**DOI:** 10.1371/journal.pone.0159910

**Published:** 2016-07-26

**Authors:** Krisna Patel, Kate Tchanturia, Amy Harrison

**Affiliations:** 1 Department of Psychology, Institute of Psychiatry, Psychology and Neuroscience, King’s College London, London, United Kingdom; 2 Department of Psychological Medicine, Section of Eating Disorders, Institute of Psychiatry, Psychology and Neuroscience, King’s College London, London, United Kingdom; 3 Regent’s University London, Faculty of Humanities, Arts and Social Sciences, School of Psychotherapy and Psychology, London, United Kingdom; 4 Ellern Mede Service for Eating Disorders, London, United Kingdom; Kyoto University, JAPAN

## Abstract

Previous research indicates adults with eating disorders (EDs) report smaller social networks, and difficulties with social functioning, alongside demonstrating difficulties recognising and regulating emotions in social contexts. Concurrently, those recovered from the illness have discussed the vital role offered by social support and interaction in their recovery. To date, little is known about the social skills and social networks of adolescents with EDs and this study aimed to conduct focus groups to explore the social functioning of 17 inpatients aged 12–17. Data were analysed using thematic analysis and six core themes were identified: group belonging, self-monitoring, social sensitivity, impact of hospitalisation, limited coping strategies and strategies for service provision. Key areas for service provision were: management of anxiety, development and/or maintenance of a social network and development of inter and intrapersonal skills. The most salient finding was that adolescents with EDs reported social difficulties which appeared to persist over and above those typically experienced at this point in the lifespan and therefore a key area for future focus is the development of appropriate coping strategies and solutions to deal with these reported difficulties.

## Introduction

Eating disorders (EDs), including anorexia nervosa (AN), bulimia nervosa (BN) are serious and complex mental illnesses which have a biopsychosocial pathogenesis and may take a chronic and disabling course. EDs not only impact physical and psychological health, but have been shown to significantly affect quality of life, relationship status and educational achievement [1]. The lifetime prevalence of AN is 0.8% and 2.6% for BN [2], with a peak age of onset between 13–18 years [3]. AN has a mortality rate of at least 5–6%, the highest of any psychiatric illness, and is described as one of the most treatment-resistant psychological illnesses [4].

Social functioning typically refers to an individual’s ability to successfully interact with their environment [including work, school, social activities and relationships with partners and family]. This is facilitated by the development of a range of social skills including verbal and non-verbal gestures, social cognition and interpersonal functioning. Although not a diagnostic criteria, data suggest that EDs are associated with atypical social and emotional functioning [5–7]. In a longitudinal Swedish community study, a strong relationship between social interaction problems and AN was reported [8]. In this cohort of early onset cases, a quarter of patients with AN who also had antecedent social-communicative problems continued to have a poor psychosocial outcome at age 35 [9]. At the same time, other studies highlight the important role of social support [10], social functioning and social inclusion [11] in successful recovery for adults with ED with the absence of these factors associated with delayed recovery [12].

In spite of the perceived importance of supportive social relationships in recovery, patients with EDs report having small social networks [13], which are of poorer quality than non-ED controls [14]. Adults with AN have been observed to be typically shy and socially withdrawn [7, 15, 16] and report engaging in fewer social leisure activities and more solitary activities [7], a pattern reported to continue into adulthood [17] and after behavioural symptoms of the ED have resolved [14] and may be related to poorer facial expressivity of emotions in social interactions and emotion regulation [18]. Interestingly, whilst social communication difficulties are often associated with delayed recovery [19] and are predictive of poorer overall outcome [9, 20–22] in adults with EDs some former inpatients have suggested that the inpatient setting provides opportunities for the formation of strong relationships with other patients who offer a source of social support to the exclusion of existing friends outside of the illness [23, 24].

Research studies have predominantly focused on exploring social functioning in adults with EDs. Of the limited research conducted on adolescents with EDs, studies have highlighted the role of adverse contact with friends, including derogatory comments, negative feedback and pressure, as triggers of EDs [25]. Adolescents with high ED pathology in a non-clinical sample reported experiencing more difficulties with friends, including conflict and alienation, and were less likely to endorse friends as a source of help and self-validation [26]. This research suggests that interpersonal adversity may be a risk factor for EDs, however it is difficult to establish a causal relationship between social functioning and EDs; with contemporary models proposing that socio-communicative difficulties are maintaining factors for patients with EDs [27, 28] via supporting distorted eating behaviours and maladaptive coping strategies.

Of the few qualitative studies of adolescents with EDs, peer support from existing friends, supported discharge and feeling connected to others have been identified as fundamental factors for recovery. These findings were synthesised in a review which highlighted the importance of maintaining peer relationships before, during and after treatment in order to maintain a sense of normality [29]. Further work suggests that maintaining positive changes following treatment was strongly related to a non-judgemental stance from family and friends, with patients acknowledging that recovery involved reconnecting with others and developing trust in others [12]. The research discussed above has highlighted some of the aspects of social functioning which may be disturbed in adolescent patients with EDs, but the evidence base remains scant and little evidence exists regarding why such difficulties might occur from the patient’s perspective.

The types of social difficulties outlined above are not unknown to adolescents without EDs, As adolescence is a unique stage in human development and typical brain maturation at this point in the lifespan is proposed to result in a sensitive period in adapting to one’s social context, leading to difficulties with social cognition. Indeed, peer acceptance, social evaluation and negative peer influences are considered adolescent-typical behaviours [30]. However, it is possible that the adolescent onset and possible effects of starvation on the brain may disrupt, delay and compound transition through the typical social developmental and neurodevelopmental changes which happen during adolescence and early adulthood, such as synaptic pruning and myelination of long tracts [31]. Changes at the neurobiological level and systemic and socio-cultural factors may act in a recursive way and maintain the cycle of ED pathology. Key risk factors for EDs such as early adverse experiences, low parental care and interpersonal adversity combined with temperamental features such as shyness and inhibition; core beliefs of powerlessness, thin-ideal internalisation, low self-value and increased anxiety may lead to a negative self-concept, shame and subsequent isolation and a reduced social network may contribute to limited social cognition and interpersonal skills [32–36]. Taking this into account, it is important to understand more about the social experiences of adolescents with EDs.

Although it is suggested that treatment should focus on resolving symptoms [37], there is an argument for addressing social recovery alongside specific eating-related issues, particularly given that patients rarely show significant improvement with regards to social impairments from admission to discharge [14]. Early intervention for EDs appears key [38, 39], however less is known about the difficulties adolescents, compared to adults, with EDs may face in terms of social functioning. This study aimed to use qualitative methodology in the context of a service audit with young people with EDs to explore participants’ experiences of social interactions, their perceptions of their social skills relative to their peers’ and the type of support young people with EDs feel services should provide around their social functioning and social recovery.

## Methods

### Participants

Participants were recruited using opportunity sampling from a specialist ED inpatient service in the UK, for individuals aged 8–18 years. The diagnosis of an ED is confirmed on admission by a Consultant Psychiatrist based on DSM-5 criteria [40]. Participants were purposively sampled and were told about the study during the weekly community meeting, where the first author disseminated information sheets detailing the study and invited patients to join the focus groups. Patients were required to have the competency or capacity (assessed by the Consultant Psychiatrist) to understand the nature of the investigation were included in the groups and individuals were required to be able to speak English to a sufficient degree to be able to participate in a conversation. Patients with any form of ED were eligible for the study but due to the nature of the service, all patients admitted at the time of recruitment had a diagnosis of the restricting or binge-purge subtypes of AN. The sample size was defined by the criterion of data saturation a guiding principle often used in qualitative research [41] such that the final sample was achieved when new information was no longer being added. Data saturation ensures that all perceptions that might be important are uncovered, without the sample becoming too large so that the data becomes repetitive and, eventually, superfluous.

This investigation received ethical approval from the City Road and Hampstead National Health Research Authority (NRES) research ethics committee Reference 12/LO/0409. The study was conducted in keeping with the principles of the Declaration of Helsinki and in keeping with local research governance guidelines. Written informed consent was collected from all patients aged 16 and above and assent was collected from those aged 15 and below; with written informed consent collected from their parents. Patients were offered a full debrief in both written and verbal formats at the end of the discussions. The above consent procedures were approved by the aforementioned ethics board. Further demographic information is provided in Table 1, below.

**Table 1:** Patient Demographics and Characteristics.

Focus Group	Patient	Age (years)	Gender	Ethnicity	Diagnosis	EDE-Q Global score	HONOSCA Score	Weight for Height (%)	Number Of Admissions	Length of stay (months)
1	1	14	Male	White-British	AN Restrictive	0.62	20	92.1	3	21
	2	17	Female	White-British	AN Restrictive	4.13	28	94.08	2	11
	3	13	Female	White-British	AN Restrictive	Unknown	28	97.21	3	18
2	4	15	Female	White-British	AN Restrictive	2.7	16	94.76	2	16
	5	12	Female	White-British	AN Restrictive	3.4	11	93.51	2	40
	*6	13	Female	Arab	AN Restrictive	5.4	17	106.72	2	6.2
	7	12	Female	White-British	AN Restrictive	3.05	12	94.35	1	9
	8	15	Female	White-Irish	AN Restrictive	4.8	17	80.53	2	6
	9	12	Female	White-British	AN Restrictive	5.3	26	81.62	4	16
3	10	15	Female	White-Irish	AN Restrictive	4.62	9	87.34	3	30
	11	15	Female	Mixed-White	AN Restrictive	2.01	7	93.95	3	14
	12	13	Female	White-British	AN Restrictive	4.62	17	97.2	1	12
4	*13	17	Female	White-British	AN Restrictive	3.07	23	78.32	1	4
	14	15	Female	Asian-Indian	AN Restrictive	0.6	Unknown	68	1	3
	15	15	Female	Asian-Indian	AN Restrictive	3.1	11	93.46	2	8
	16	13	Female	White-British	AN Restrictive	4.33	30	77.9	2	6
	*17	16	Female	White-British	AN Restrictive	4.6	21	81.62	3	8.2

EDE-Q, Eating Disorder Examination Questionnaire (Fairburn & Belglin, 1994); HONOSCA, Health of the Nation Outcome Scales Child and Adolescent Mental Health (Gowers et al., 1998). N.B. HONOSCA scores, weight for height measurements and length of stay were calculated on the day of data collection by an assistant psychologist using the following formula: (child’s weight/reference weight for child of the same height)*100; EDE-Q scores were taken on day of admission by a clinical psychologist. Where ‘unknown’ is stated, there was no data available for the patient. Patients were informed and consented to this information being accessed by authors. Length of stay refers to inpatient admission in any ED service. Number of admissions refers to the number of times the patient has been admitted into any ED service during their illness and is an approximation.

*Participants were present during the focus group but were interviewed individually. Weight for height percentages are reported to provide a measure of expected body weight and are used in place of body mass index as they are more sensitive to child and adolescent populations.

### Measures

A semi-structured topic guide was developed based on the literature search (see Table 2), with open-ended questions selected in order to encourage an unbiased and open discussion among participants. For validation, questions were presented to a group of informants (two experts in the field of EDs) and two patients due to be discharged from the service to check for appropriate phrasing, content and understanding.

**Table 2:** Semi-structured questions developed for the focus groups.

What are the sorts of problems you have had with friends in the past?
In a social situation what are the things that worry you the most?
Why are some people more successful at parties than others?
How has coming to hospital affected your social lives?
What would you need from the hospital to help you feel more comfortable in social situations?

### Procedure

Qualitative methodology allows for a deeper understanding of the meaning of a persons lived experiences, behaviours, feelings and emotions [42]; factors difficult to capture using quantitative methods [43]. To elicit participants’ experiences and ideas, four focus groups were held. Focus groups were used as patients were already familiar with discussing sensitive issues in group settings and this was thought to provide a more comfortable setting for patients. In this patient group, research indicates that a focus group scaffolds patients and thus allows individuals to discuss topics more easily [44]. Each focus group was voluntary and lasted 30–45 minutes. Focus groups were divided according to the ward patients were admitted on (two groups per ward) due to familiarity with the other patients. Ward one (participants 1–9) provides a rapid response service with intensive nursing intervention and typically admits patients with a co-morbid psychiatric disorder alongside the ED. Ward two (participants 9–17) offers intensive treatment for severe EDs.

Typical duration of stay is 6 months, during which time patients spend increasing periods of time at home and reintegrating into school/college. Patients participate in a structured daily programme of therapeutic sessions and education at an onsite school. Treatment primarily focuses on nutritional rehabilitation and patients are offered individual therapy, group-based Cognitive Behavioural Therapy (CBT), occupational therapy and family therapy.

Each group was facilitated by the first (KP) and moderated by the last author (AH). During the focus groups, it was observed that a small number of patients found it difficult to express themselves in the group setting (e.g. they did not answer any questions) and a decision was taken by the first author to approach these individuals for a one-to-one interview (using the same questions) to avoid excluding their views. In total, three individual interviews were conducted (patients 6, 13 and 17), facilitated by the primary investigator (KP). No demographic or characteristic differences were noted between focus group or interview participants. All focus groups and interviews were recorded using a digital dictation machine and the discussions were transcribed verbatim for analysis; any details were anonymised to preserve the identity of participants. Field notes were taken to record events and perceptions throughout data collection.

### Data Analysis

Thematic analysis [45] is a technique to analyse qualitative data by distilling themes and disseminating findings. It was considered suitable because of its flexibility, theoretical freedom and its descriptive rather than interpretative function, making it preferable to other methods, such as Interpretative Phenomenological Analysis (IPA); [46] and Grounded Theory [47]. Whilst IPA focusses on detailed systematic interpretation of personal experience and Grounded Theory uses theoretical sampling to develop a theoretical explanation, thematic analysis focusses on the description of divergence and convergence of experiences.

A hybrid process of inductive and deductive thematic analysis of raw data was utilised in that some themes were identified from the data whilst others were hypothesised a priori.

To capture any potential differences between wards, all focus group and interview transcripts were divided by the ward they were conducted on. Primary analysis was undertaken by one author (KP). There was a strong homogenous trend with regards to themes, with all themes recurring across all participants in both wards. The transcript was repeatedly examined in depth and coded for the presence of references to social skills and functioning. The preliminary themes were continuously reviewed for salience and importance and for clustering by content. This iterative method of constant comparison was used in order to reduce and condense the themes into the most salient categories. Subordinate themes were first established for each question, and then cross–validated, adjusting if required. Superordinate themes were chosen on the basis of forming ‘umbrella’ themes that covered various subordinate themes.

Validation process and reliability standards for the conduct of good qualitative research as delineated by Henwood and Pidgeon [48] and Elliott, Fischer and Rennie [49] were followed in this audit. Triangulation is a method often used in qualitative research to corroborate findings to increase reliability and validity of results, ensuring that accounts are rich, robust and comprehensive. It was pursued by combining different sources of information (e.g. understanding the research question from multiple perspectives), having a facilitator and moderator present during groups to adequately observe group dynamics and behaviour and multiple analysts. Using this technique, 20% of the data were also analysed by a co-author (AH) and joint discussions were held to ensure that codes and themes were adequately grounded in the raw data. In addition, a reflective diary was maintained to ensure transparency and credibility of the themes identified; the master table of themes was continuously updated throughout analysis; the identification and inclusion of contradictory or negative cases and accounts were sought; participant theme validation was pursued through offering participants transcripts and drafts of the thematic analysis throughout the preparation of the report; and the findings of the research were presented to patients and staff.

In line with this type of qualitative approach, a reflective summary is provided at the end of the discussion.

## Results

Of 26 patients admitted to the service at the time of recruitment, (21 with a diagnosis of restricting AN, two with a diagnosis of binge-purge subtype AN and three with a diagnosis of pervasive arousal withdrawal syndrome), 14 (one male; 7%) agreed to participate in the focus groups and 3 agreed to participate through individual interviews. Six patients chose not to participate due to a lack of interest and three were unable to provide informed consent. Although all patients regardless of diagnosis were invited to participate in this study, this study cohort consisted of those diagnosed with AN restrictive subtype. There was a high level of congruence across focus groups and individual interviews, with six core themes identified: group belonging, self-monitoring, social sensitivity, limited coping strategies, two of which were hypothesised a priori: impact of hospitalisation and suggestions for service provision (see Table 3, below).

**Table 3:** Themes identified from the focus groups and interviews.

*Theme*	*Definition*
*Group belonging*	A sense of fitting into their social network and being a part of group knowledge.
*Self-monitoring*	Close regulation of communication and behaviour to accommodate and manage their social environment.
*Social Sensitivity*	An increased likelihood of perceiving criticism and of experiencing negative affect to perceived criticism.
*Limited coping strategies*	An absence of solutions and strategies to overcome difficulties in social skills and functioning.
*Impact of hospitalisation*	Positive and negative factors of hospitalisation that have contributed to the patients social functioning.
*Suggestions for service provision*	Potential contributions and interventions the ED service can provide to support social engagement in patients.

All superordinate and subordinate themes were found to be intricately linked, discovered and illustrated via conceptual maps during the analysis process. The following section will present the key themes identified in the data and use excerpts from the transcripts to illustrate the themes.

### Superordinate Theme 1: Group Belonging

**Fig 1:**
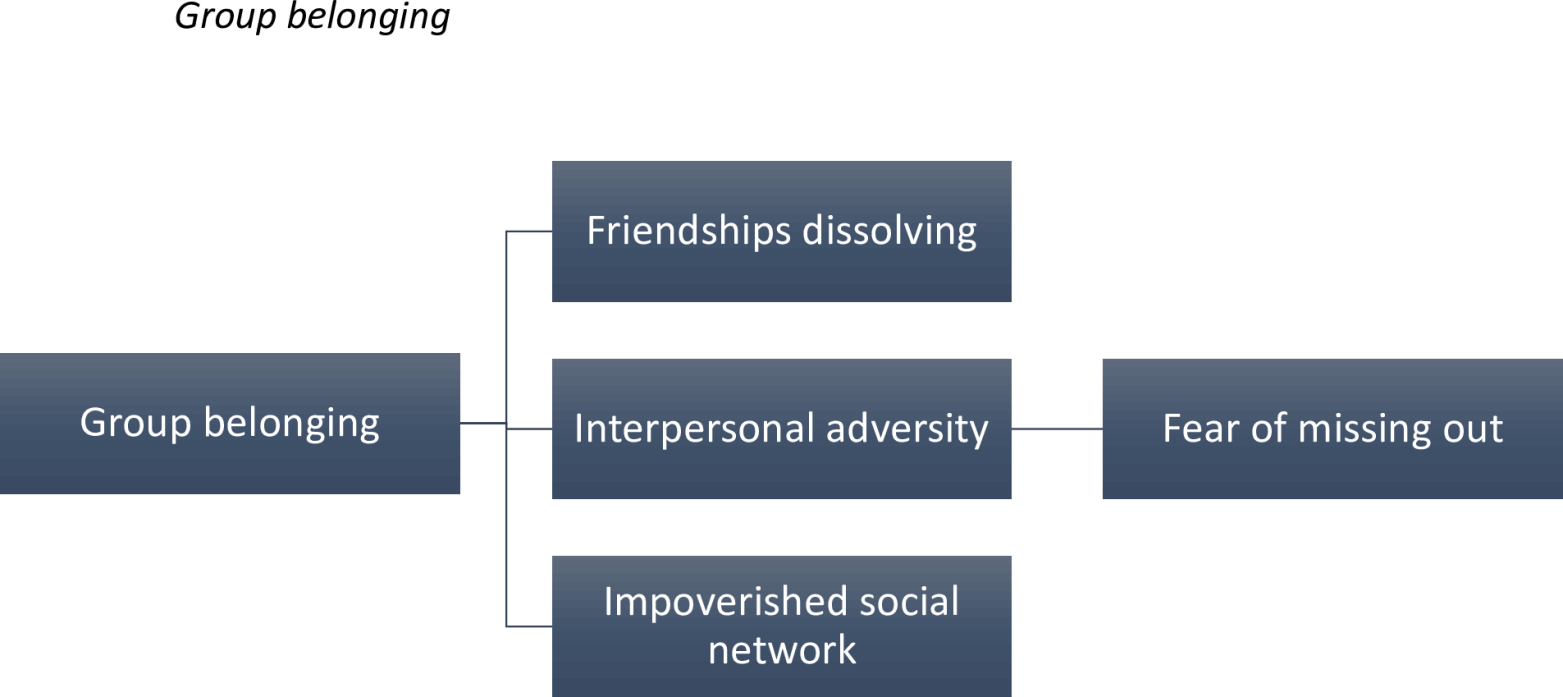
The furthest left hand box indicates the superordinate themes, with the subordinate themes in the remaining boxes. The connecting lines indicate the hierarchical organisation of the themes.

The theme of group belonging comprised of three subordinate, interrelated constructs: friendships dissolving, interpersonal adversity and impoverished social networks (see Fig 1).

#### Friendships dissolving

Patients reported reduced social capital, in that they either did not have friends prior to admission or they lost friends in the process of illness. Many felt that this was due to losing connection and not knowing what to say anymore. Some patients took responsibility in their friendships dissolving in that they described that they had chosen to move on, change interests or had outgrown friends. However, many responses were marked by a sense of loneliness, isolation and abandonment, in that their reality was now different to their friends:

“I mean in this context it’s difficult to have time for people. I’ve spent the majority of my time like in hospitals and in different medical appointments and I literally would never have time to see anyone. I think it’s hard for them to just understand the whole situation like the last time I saw my like best friends I was in general hospital and its quite hard to explain it to them like what’s going on and then they’re all just like when are you coming home, when are you out and you can’t really give them a date or anything.”

The very nature of an inpatient setting created a sense of feeling removed from the outside world and from normality. Patients frequently reported feeling as though life in hospital was static and life on the outside for friends was dynamic, with long periods of absence affecting their friendships:

“They still have their life to carry on with… It’s kind of like I dunno. It’s like your life is on hold, but it’s not, because when you get out your just it all changes so much and I guess like when you go out all of your friends will have moved on and you’ll have missed quite a big chunk of what’s happened.”

Friendships dissolving were attributed to repeated admission and consequent restrictions and difficulty in maintaining contact. Being an inpatient for an extended period led to the feeling that friends had forgotten and abandoned them. This was especially poignant for patients attending hospital a long distance from home, who noted difficulties in both maintaining old friendships and establishing new ones, and who noted the impact hospitalisation had on family dynamics. Patients reported being too far away from current friends for frequent visits and being unable to establish new friendships due to ward restrictions and then potential discharge:

“It’s hard though because like this is nowhere near where I live so I would go here and make friends and then move and then when I go home I won’t have them coz I live like two to three hours away so it’s quite a way”

#### Interpersonal adversity

Interpersonal adversity includes teasing, bullying and competitiveness. Patients felt that the experience and resolution of adversity was different for boys and girls; for girls, it led to social exclusion and could last indefinitely, whereas for boys, physical altercations were reportedly used to often resolve problems. Teasing often escalated into gossip and rumours, and was linked to a reduction in self-worth and confidence:

“Literally, you thought someone was your friend and the next day you’d find out and they’d be like no so and so said this, you said this and you said that and oh my god everything gets twisted and then bam you get a massive bitch fight.”

“With my experience with an eating disorder like I remember I came out of hospital last time and when I went back to school they would say the wrong thing to me and also like it wasn’t just friends it was like people would pick on me as well and lie.”

As a means of coping and in an effort to *“fit in”* and *“not stand out”*, patients described feeling pressure to take part in illegal activities. Not partaking led to fear of judgement, social rejection and further bullying and teasing:

“… not wanting to stand out and trying to fit in … some of it is like trying to fit in with your friends but also like if they’re doing something wrong and you know it’s wrong and you don’t wanna do it but you don’t wanna like stand out.”

Fear of missing out was also discussed in the context of interpersonal adversity, with feeling left out of friendship circles and events compounding the sense of exclusion and rejection. Many patients described a fear of missing out on social opportunities both within school (e.g. school plays) and outside, and of missing out on being a part of group knowledge. This was related to both the ED and being hospitalised (and the subsequent physical separation and not being able to leave the ward), and to other factors such as a loss of connection with friends, self-isolation and deliberate exclusion from social group:

“… and you feel like you’re not in the loop … yeah you feel like you’re missing out … coz life’s going on without you.”

#### Impoverished social network

The result of friendships dissolving, interpersonal adversity and fear of missing out was related to a reduction in the size of patients’ social networks. This was noted in the context of illness related factors, such as hospitalisation and self-isolation, and also in relation to the impact of this on future interactions, in that it led patients to believe that they were no longer privy to group knowledge, shared the same interests or had the same social opportunities after discharge:

“Yeah being in hospital affects a lot to do with your social network. You can’t really keep in contact with your other friends it’s just really hard. You don’t have any internet which is mostly where you would see your other friends.”

*“What social life*? (laughs) *You know*, *I haven’t had a social life for so long I’ve forgotten what a social life is*, *my social life in here is non-existent*.*”*

### Superordinate Theme 2: Self-Monitoring

**Fig 2:**
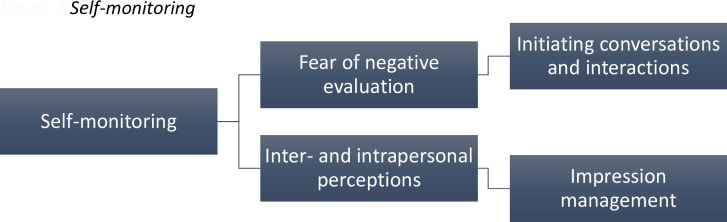
The furthest left hand box indicates the superordinate themes, with the subordinate themes in the remaining boxes. The connecting lines indicate the hierarchical organisation of the themes.

The theme of self-monitoring had two subordinate themes: fear of negative evaluation discussed in connection to initiating conversations and interactions and inter and intrapersonal perceptions related to impression management (see Fig 2).

#### Fear of negative evaluation

Others’ perceptions were hugely important to patients and this was one of the first topics mentioned when asked about social situations. The most prominent external source of uncertainty was the reaction of others in interpersonal encounters (reaction to self and to the illness), which led to excessive monitoring of appearance:

“What people will say about me I get worried about that like if they make a comment about my appearance or my weight or my shape I get worried about that especially when I have known somebody for a long time. I get really conscious around people as well like I’m scared that they might judge me and stuff like that even if I don’t know them.”

Fear of negative evaluation led to excessive rumination about what to say and how to act, with patients avoiding spontaneous communication and often withholding thoughts and feelings:

“I dunno might have something to say but they’re just afraid, afraid like if they say, I dunno, what people would think of them.”

Participants described sensitivity to perceived negative affect from others, particularly fearing that once they have left a social encounter they would be mimicked, talked about and judged. Although most patients expressed concern about dealing with negative affect, accepting compliments was also a frequently reported concern. Often patients described feeling suspicious of others and their intent:

“I’m always thinking that they are judging me. Like what are they actually thinking? Behind their, behind that face of theirs, what are they actually thinking?”

Judgement from others was a consistent fear amongst patients, both in relation to a wide array of ED (weight, image) and non-ED (academic achievement, intelligence) factors. It was one of the most frequently reported fears of being in a social situation, and patients were very sensitive to any form of perceived or actual judgement. Perceived judgement was described as heavily impacting interactions and motivation to seek contact:

“I’m scared of being judged by other people so yeah I just tend to keep myself to myself.”

“You don’t know what like, you might say something and they’ll just judge you straight away on what you say.”

Patients felt this fear of judgement stemmed from an individual’s temperament and noted that introversion (shyness, self-isolation) compounded this fear, resulting in patients prioritising other activities such as work and academia at the expense of social functioning. Conversely, patients discussed that extroverts were less likely to fear judgement and were linked to more social success, in that they were more likely to experience social engagements and activities, display risk taking behaviour, be *“loud and chatty”* and were less likely to be inhibited. This temperament was often linked to popularity and impressing people, and therefore social success.

#### Inter and intrapersonal perception

Self-monitoring seemed to mediate both inter and intrapersonal perceptions in both verbal communication and motivation to seek contact: being stuck for what to say, afraid that they don’t have all the answers or making sure they didn’t say the wrong thing:

“Like what to say, coz sometimes you might say the wrong thing and then it might be awkward.”

Difficulty with intrapersonal perceptions was linked to ED symptomology and temperament and led to maladaptive coping behaviours:

“Erm, I’m very shy. I’m not good at talking to new people erm I have a tendency to hide erm I have a problem where I like I find it hard to make eye contact and sometimes I’ll cover my face quite a lot when I’m around people. I’m just not very good at it.”

### Superordinate Theme 3: Social Sensitivity

**Fig 3:**
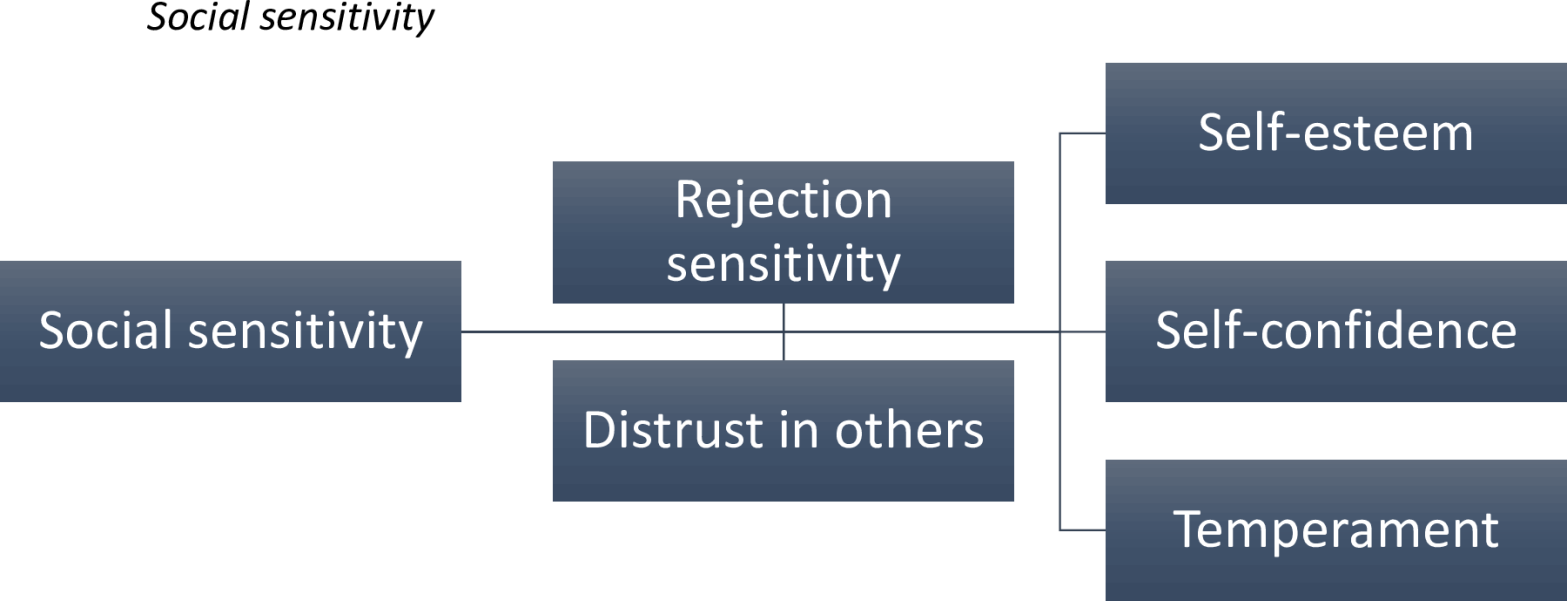
The furthest left hand box indicates the superordinate themes, with the subordinate themes in the remaining boxes. The connecting lines indicate the hierarchical organisation of the themes.

The theme of social sensitivity was related to an increased likelihood of perceiving criticism and of experiencing negative affect to perceived criticism. Two subordinate themes were identified: rejection sensitivity and distrust in others. These themes in turn were explicitly linked to self-esteem, self-confidence and temperament, both as protective (i.e. improves social skills) and predisposing factors for social difficulties (see Fig 3).

#### Rejection sensitivity

Patients tended to identify the self in appearance-based objectified terms, as opposed to competency-based terms, which they often downplayed. Idealised values about the importance of appearance were consistent across all groups. Patients noted being sensitive to any form of perceived rejection, whether that be appearance or behaviour based. The anticipation of rejection often led to feeling socially anxious.

“…It’s important like how they look like if they’re good looking it increases their confidence…(it matters what) people (think) because like er it’s like you might not realise that you’re good looking but when someone tells you that you’re good looking you get confidence yeah.”

#### Distrust in others

Closely linked with suspiciousness was the concept of trusting others, chiefly around disclosing the illness and hospitalisation without being judged, but also generally trusting people to be themselves:

“People are so fake like they pretend to your face that they like you and then the next minute you know they’re like saying all sorts of behind your back.”

### Superordinate Theme 4: Limited Coping Strategies

**Fig 4:**
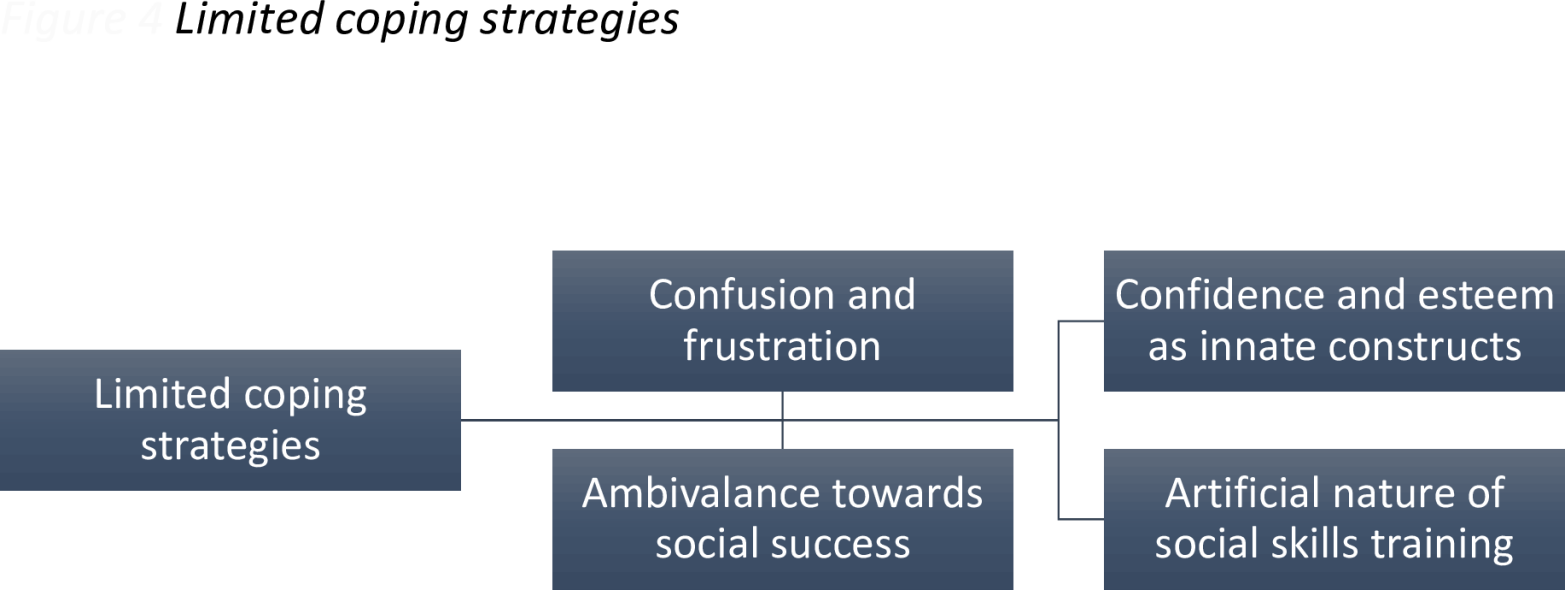
The furthest left hand box indicates the superordinate themes, with the subordinate themes in the remaining boxes. The connecting lines indicate the hierarchical organisation of the themes.

The theme of limited coping strategies was linked to several subthemes (see Fig 4). What is unique about this theme is that it arose predominantly from observation of a distinct lack of discussion, rather than something explicitly referred to by patients. What was most striking was that patients showed disinterest and ambivalence towards social success and strategies to achieve it, field notes indicated patients rolled their eyes, physically turned away (wrapped themselves in blankets and faced the inside of the sofa) and became distracted with their environment (e.g. picked up a book, played with loom bands) when asked about social success in others. The majority of patients struggled to understand what was being asked of them and to think of ideas and solutions around the sorts of ways they might manage social situations themselves. The question regarding what makes some people more successful in a social situation was frequently met with frustration and confusion. Patients expressed not caring about social success or frustration over what the question was asking and many described *“just not getting it”*. The question was met with silence, a general confused response or an expectation that someone else would know the answer: *“I don’t*, *that’s a silly question”*, *“I don’t know how to answer that question”*, *“Don’t look at me neither do I”*. It is possible the question was poorly worded although even when the question was elaborated on or rephrased, patients remained unclear. Although patients were able to think of why others might be more successful in social situations, they themselves were unable to think of strategies to overcome some of the difficulties that they had themselves described. Patients stated, for example, that others seemed to do better because they were *“more confident and had higher self-esteem”* and although they were able to elaborate to some degree what “confidence” might consist of, they were unable to state how this may be achieved and referred to them being innate constructs that led to better social skills rather than something that can be learnt:

“It depends if the person’s confident or not like if the person’s confident they’ll be able to speak to other people and they be able to have a fun time but if they haven’t got much confidence I think it would be quite difficult for that person to try and make a conversation with someone else. Some people just, they fit in.”

Whilst able to recognise that they had negative thoughts that led to distress, avoidance and anxiety, patients were unable to identify solutions to better manage them. Interestingly, whilst patients frequently described what would be maladaptive coping behaviours when referring to potential worries in a social situation (withdrawal and isolation), they were unable to come up with other ways to solve the problem. Similarly, whilst all patients reported some form of adversity, none were able to describe methods to resolve problems or suggest how they may obtain help. Often they described resorting to participating in illegal activities in a bid to belong and reduce bullying, but couldn’t describe other strategies that they might employ. Whilst patients were able to describe the values of friendship, they were unsure about ways to elicit support or even how to form friendships. One patient noted the potentially artificial nature of social skills training in this context, but couldn’t think of another way friendships could be achieved:

*“There’s not really anything they can do coz they can’t ring up people outside and say right when (…) comes out of hospital can you be her friend*. *It’s* (laughs) *not gonna happen*. *I dunno coz you can really teach someone how to behave in a social situation coz then it just makes it awkward coz it’s like staged*.*”*

### Superordinate Theme 5: Impact of Hospitalisation

**Fig 5:**
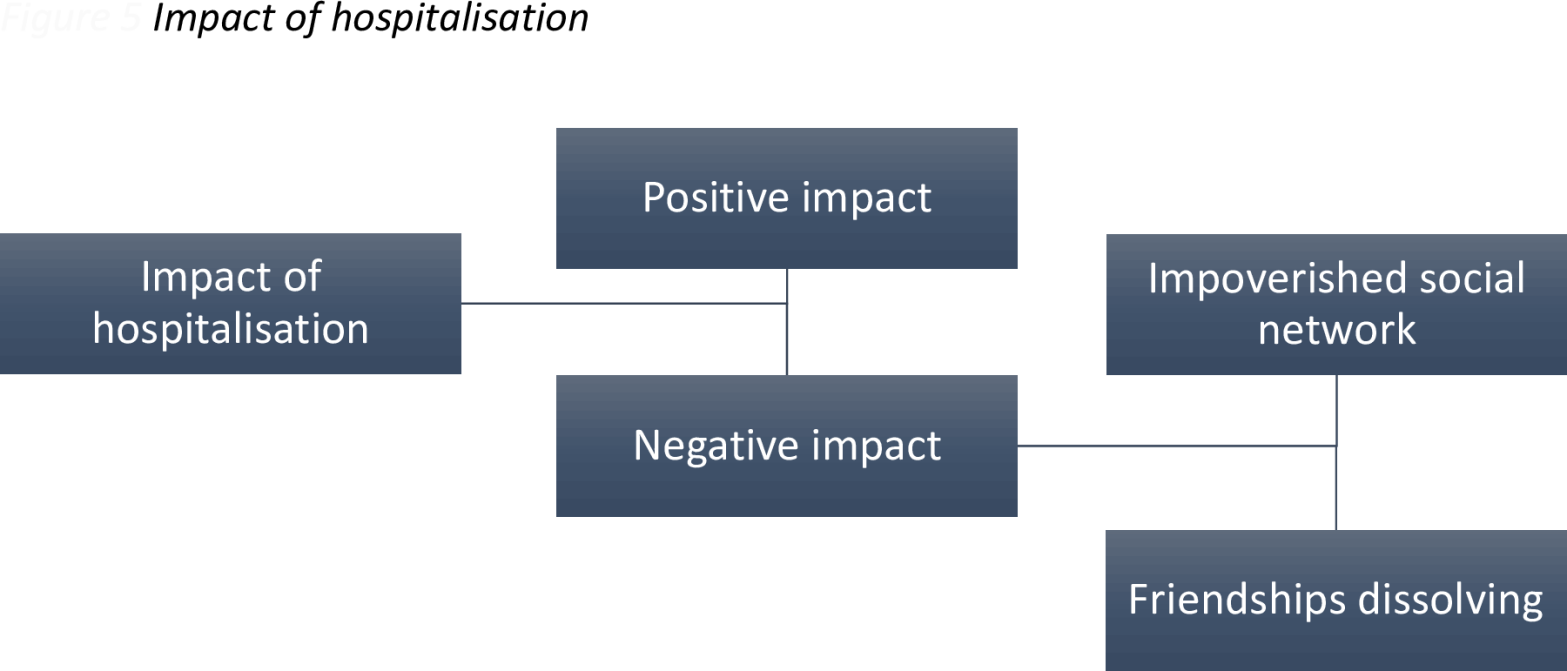
The furthest left hand box indicates the superordinate themes, with the subordinate themes in the remaining boxes. The connecting lines indicate the hierarchical organisation of the themes.

The patients identified both positive and negative aspects of hospitalisation (see Fig 5). Negative impact was closely linked to an impoverished social network and friendships dissolving.

#### Positive impact

Patients identified several positive aspects of hospitalisation on their social functioning, particularly having a social network made up of other people with EDs resulting in a safe setting. Individuals described how certain aspects of hospitalisation (e.g. group activities and therapy) led to opportunities to meet different people, form friendships quickly and increase trust and confidence:

“Because you are around people twenty-four hours a day so you kind of form closer friendships quicker”

Some patients noted that being hospitalised increased their trust and bond with friends outside of the service, particularly as they showed consistent support after disclosing the illness and patients did not feel judged when they did so:

“With my close close friends, like proper best friend, I feel like we’ve built more of trust, because they stuck by you through the hard time, and you know that they’re there and they listen to you as well.”

#### Negative impact

However, patients noted that the connection with friends/family outside of the service decreased as they relied upon fellow patients for support, friendship and social activities and recognised this was a way of potentially staying in the ED:

“I think also sometimes you find it easier to stick with er when you come into hospitals anyway you find it easier to stick with and make friends with the people who are have illnesses as well I guess, it takes you away a bit from your friends and I think that’s you trying to stay around the illness as well so it can be difficult to differentiate.”

The negative impact of hospitalisation far outweighed the positive, particularly around reducing an already depleted social network and contributing to friendships dissolving.

#### Impoverished social network

Patients reported that restricted activities and limited use of mobiles and computers further distanced them from their social network, and the means by which they would normally communicate with friends. Sharing something in common seemed an important factor to establishing friendship, as iterated throughout the focus groups/interviews, and patients expressed concern that being in hospital restricted this and led to the ED becoming the common interest. Infrequent or sporadic contact with friends on the outside was also reported to impact access to peer support, both emotionally and with academic work, leading to loneliness, isolation and a sense of falling behind in both respects:

“It’s very hard to speak to people and obviously I don’t have a phone, can’t use Facebook or any social networks and erm even when I do use the main phone but you can’t have very personal conversations.”

### Superordinate Theme 6: Suggestions for Service Provision

**Fig 6:**
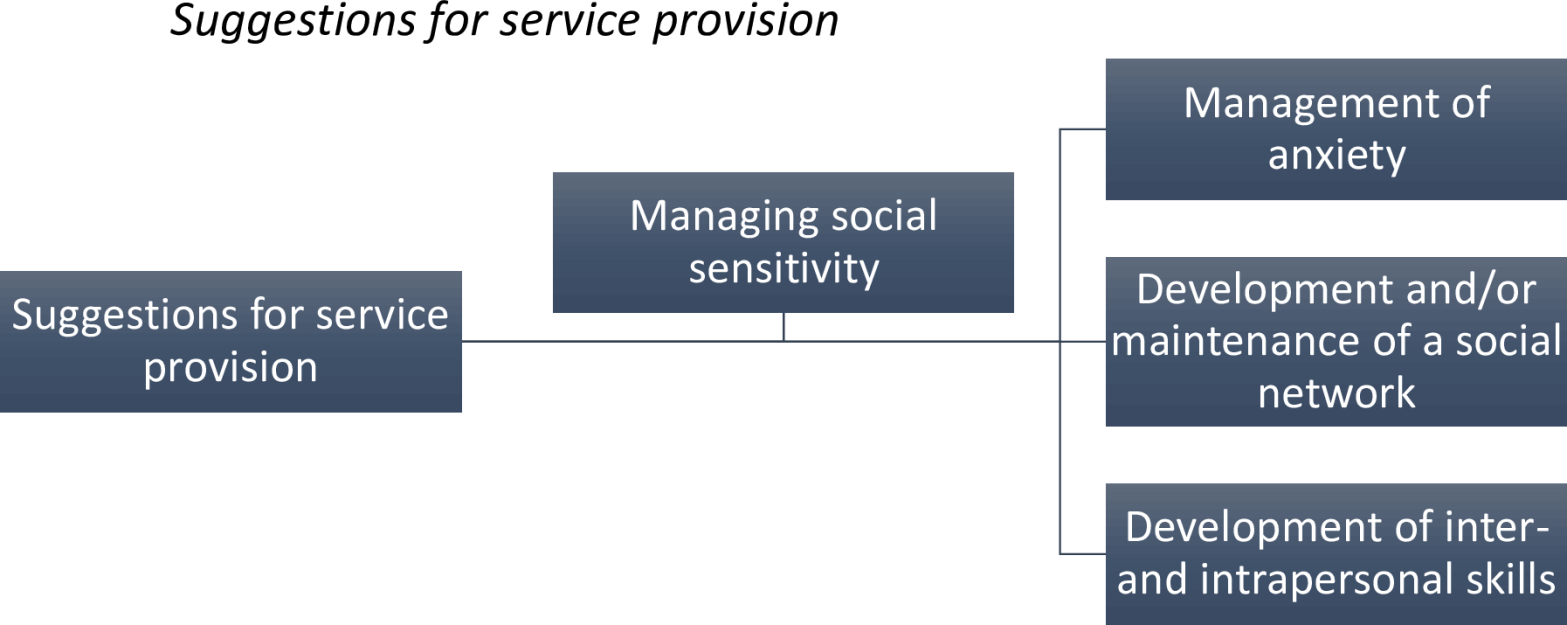
The furthest left hand box indicates the superordinate themes, with the subordinate themes in the remaining boxes. The connecting lines indicate the hierarchical organisation of the themes.

When asked what the service could provide for the patients with regards to social skills and functioning, patients mentioned: management of anxiety, development and/or maintenance of a social network, and development of inter and intrapersonal skills (see Fig 6).

#### Management of anxiety

Adolescents requested more opportunities to connect with *“normality*” outside of hospital and stepped integration back into the community to help manage social sensitivity. This ranged from having meals around other people and developing techniques to deal with perceived criticism and negative affect from others to taking walks during busy periods to more home/school leave.

“Maybe like do things gradually like if you’re going back to school do it like in small steps and not like straight away so it’s not too overwhelming.”

Some patients described wanting to learn mindfulness and meditation techniques to reduce anxiety in public:

“… coz when you are like in a crowded place you can’t concentrate coz if you meditate after a while you get better so you would get like you can concentrate more”

Patients described how strict provisions around privacy prevented them from building a relationship and having sensitive conversations with others, and although they understood why they were in place, they noted the impact this had on trust:

“I know there’s like a rule that you’re not allowed in your bedroom with other people unless you’re both supervised and I understand the meaning for that but like sometimes you don’t wanna like you just want some time with someone. Staff all around you feel you can’t really have a chat with your friends.”

#### Development and/or maintenance of a social network

The return to school and social lives was seen as daunting and patients requested support regarding what to say to people about where they have been and whom to tell, how to cope and how to involve their close friends as a means of support. Patients requested more social interactions between the wards, increased use of social media, use of pen pals, encouraging hobbies to develop common interests and increased visitation from friends:

“I think they should encourage more visits with like closer connections with friends with established connections like that before leaving. It would help them understand you and what is going on.”

Some patients felt there were inflexible rules within the service, with little regard for unique patient characteristics and preferences, for example wanting to be alone occasionally. Patients also described wanting staff to acknowledge the difficulty in interacting with different age groups and its potential impact on interacting with their own age group after discharge

#### Development of inter and intrapersonal skills

This theme was discussed frequently with reference to reducing negative self-perception and dealing with negative comments. Some patients wanted to look beyond their time in hospital and wanted support in increasing independence, for example via skills for employment or parental independence. Others wanted to focus on more immediate development of interpersonal skills to fit in and increase confidence in social interactions:

“Well like if something’s said to you how you could find something to say back like without I dunno how to even put this like if someone makes a comment about how, where you’ve been or something to find an alternative to say something without it causing a big deal about things so you don’t get so stressed out about it when you do eventually leave.”

## Discussion

The aim of this investigation was to explore social skills and functioning in young people with EDs, consulting with service-users on how services can support their social recovery. Six core themes were identified: group belonging, self-monitoring, social sensitivity, limited coping strategies, with the remaining two themes hypothesis a priori: the impact of hospitalisation and suggestions for service provision. Three key interventions were identified for service provision: management of anxiety, development and/or maintenance of a social network and development of inter and intrapersonal skills. These interventions were closely related to the social functioning difficulties the patients described. The identification these core themes illustrates the factors that may underline some of the difficulties in social skills and functioning that contribute to the development and maintenance of EDs. The themes are discussed below in the context of current literature.

**Group belonging: **In adolescence, difficulties developing peer relationships are common [30], and group belonging is necessary for positive adjustment [50]. In patients with EDs, group belonging was described as poor, and many patients noted not having friends or losing connections prior to, or as a result of hospitalisation. These results are consistent with earlier reports on friendship experiences in adult patients with EDs [13]. The formation and maintenance of friendship requires several skills, including empathising, understanding and considering another’s perspective, verbalising feelings of self and others and engaging in efficient strategies for conflict resolution [51], skills shown to be compromised in people with EDs [52]. Given that the reported difficulties were experienced as more problematic than those experienced by peers at the same point in the lifespan, it will be important for clinicians to consider the development of these skills in their interventions.

Similarly to previous qualitative reports in adults with EDs [13, 44], adolescent patients in this study also described how pressure from illness and hospitalisation had obstructed their social life, depleting their social network. Patients described how their limited social skills made the management of everyday life difficult, resulting in fear of and lack of spontaneity in interactions with others. A strong finding related to interpersonal barriers was mistrust. Many patients described difficulties disclosing their illness or discussing their feelings and experiences, making them less likely to seek social contact, which automatically decreased opportunities to form and maintain friendships. Contrary to Doris et al’s suggestion that electronic contact increased to avoid face-to-face contact [13], patients in this study felt electronic communication was the only way to maintain contact due to the hospital location and confines of their symptoms. Some patients noted friendships became less important as the ED progressed, and this was often patient initiated (self-isolation). This difference in findings may be due to age and management technique as Doris el al’s study participants were adult outpatients and points to a potentially significant difference in the role of electronic communication in supporting social interaction in adults and adolescents and inpatient and outpatient settings

**Self-monitoring: **Self-silencing and social anxiety were prominent subordinate themes and have been previously reported in quantitative studies with adults with AN [53, 54]. Uniquely in this study along with identifying self-silencing and self-monitoring behaviours such as excessive rumination and repression of thoughts and feelings and checking behaviours we uncovered why patients felt the need to use these behaviours; primarily to combat perceived negative evaluation and to mediate inter and intrapersonal communication. As use of these maladaptive coping strategies was explicitly linked to anxious avoidance of social situations; addressing these factors in the context of shyness may have important clinical implications.

**Social-sensitivity: **In line with quantitative findings reported by Harrison, Tchanturia and Treasure [55] and Cardi, Matteo, Corfield and Treasure [56] both in adult ED patients, the adolescent patients in this study provided a qualitative account of attentional bias towards social rejection. In line with previous research, patients described fear of rejection, criticism [57, 58] and negative evaluation [59–62] leading to social anxiety and interpersonal sensitivity, and interestingly this was greater than that felt by typically developing peers [63, 64], particularly when making friends, attending social events or initiating conversation [65].

Corroborating Claes et al’s [57] findings, patients described appearance-related anxiety, which either stemmed from, or led to mistrust of others, hypervigilence to signs of interpersonal threat and suspected ill intent. Linked to this was fear of judgement, related to both ED and non-ED features, also previously reported in this population [66]. Many patients described judgement as either preventing or contributing to anxiety around social functioning. Negative social evaluation and sensitivity to rejection are both proposed as key causal and maintaining factors in ED [67] and maintaining change following treatment is associated with support, a non-judgemental stance, letting people in and trust [12]. Patients reported difficulties with all or many of these skills prior to and after the onset of their ED and although it is difficult to infer causality, this data suggests that including a focus on overcoming fear of evaluation, addressing and managing interpersonal sensitivity and issues of trust may be important in both prevention and treatment programs for ED.

**Limited coping strategies: **Repeated exposure to a psychosocial situation and inadequate alternative coping behaviours may have played a key role in maintaining the disorder, supported by the striking finding of limited coping strategies discussed by patients relative to their point in the lifespan. Findings are in line with previous reports of a difference in coping strategies amongst adult ED patients and HC, including avoidance [68, 69] and a decreased likelihood to respond actively by solving or rethinking the problem to change the situation [69, 70]. Similar to findings reported by Hutchinson and Rapee [25] and Sharpe et al. [26] from nonclinical samples, patients in this clinical sample reported that adverse events led to defensive distancing. Aversive contact was highlighted as both a predisposing and maintaining factor, as conflicts amongst friends (including gossiping and rumours) led to social exclusion and alienation and reliance on ED behaviours as coping strategies. Patients were unable to describe where or who to turn to for support and described isolating themselves as a way of coping. Reflecting Sternheim et al’s [66] findings in adult patients with ED who were able to describe optimal solutions, but struggled with personal solutions. Similarly, although constructs like self-esteem, self-worth and confidence were frequently related to social functioning, patients could not report how these constructs were acquired, and instead suggested that they were innate traits. Given these consistent findings, therapeutic interventions and early intervention programmes may need to target social problem-solving skills (e.g. resilience).

**Impact of hospitalisation: **In this inpatient setting, adolescents struggled to maintain social roles established on the outside and hospitalisation acted as an unintentional physical and emotional barrier preventing the implementation of social skills. The inpatient environment became a safe setting where patients were surrounded by those with similar difficulties and attitudes and where the likelihood of spontaneous and unexpected interactions was limited, further reducing the motivation and opportunity to practice social skills outside of the ward. This is in line with the assertion of Treasure, Crane, McKnight, Buchanan and Wolfe [71] of isolation as an iatrogenic factor and this may be particularly true for cases of repeated hospitalisation. Iatrogenic- and self-isolation could be perpetuated by difficulties in emotion recognition, regulation and expressivity [18]; given its proposed impact on recovery further research is required to delineate factors that contribute to its inception and perpetuation in adolescents with ED.

Interestingly, patients in this study also described the therapeutic value of hospitalisation in terms of expanding social relations, via the formation of close bonds with others (other patients and/or friends outside), interacting with individuals from different backgrounds, learning to form friendships quickly and trusting others; however they also noted that this social support was only available when they were on the unit and acknowledged that the risk is that this support diminishes once their ED-peers or they are discharged. Unfortunately, increased support from ED-peers came at the expense of non-ED peers with patients describing contact with friends/family decreasing as they relied on other patients for support, friendship and social activities, with such contact also described as a way of maintaining the illness. It can be proposed that at the height of their illness, establishing and maintaining social support is not a priority, patients in this study noted that as they begun to recover and particularly leading up to leave or discharge their perceptions of the need for social support beyond the unit (from non-ED peers) became more apparent, and is perhaps something worth considering as part of their treatment programme.

Data suggests that although patients may experience social support during hospitalisation, it may be at a superficial level and indeed temporary inpatient care is more likely to compound isolation and have a detrimental effect on social support from non-ED peers upon discharge, and therefore service provision should include strategies to moderate this. This data also suggests that patient’s perceptions of the type of social support they need changes over their inpatient stay and recovery, where at first they may rely and seek support from ED-peers this can only be temporary due to the nature of treatment and often comes at the expense of relationships with non-ED peers. Future research may focus specifically on delineating the types and differences in support from each social group (ED vs. non-ED peers).

Communication with those outside of the unit was also discussed in reference to access to phone calls, internet and social media sites. Increased use of social media was an intervention patients highlighted as helpful in order to maintain contact with non-ED peers. However, hospital restrictions and safeguarding (for example, to protect against the use of pro-anorexia/bulimia websites) means internet usage is to be, understandably, heavily regulated. This paper does provide evidence to ‘open’ clinical division in which contacts with the outside can be maintained; however it also acknowledges the caveats of doing so including the potential for electronic communication to act as a substitute for offline skills and hinder healthy social development by encouraging physical isolation. Services may wish to consider how these social interaction tools could be safely implemented during admission and encourage the provision of systematic guidance on social media usage and its potential benefits and caveats on ED recovery, particularly as adolescent patients are likely to use this medium of communication upon discharge or during leave. Further research specifically looking at the role of social media as a tool to aid social recovery is required to clarify the issue.

### Strategies for Service Provision: Clinical implications

The patients discussed three key interventions: management of anxiety, development and/or maintenance of a social network and development of inter and intrapersonal skills. Patients suggested increasing the frequency of contact with friends and family, stepped integration into the community and school, mealtimes with friends, and leaving the unit during busy periods. As social difficulties led to anxious avoidance of social situations, developing management techniques such as those requested by the patients (mindfulness and meditation) along with interventions to address the source of anxiety and what to do as it is happening are particularly relevant. Another key area for intervention requested by patients was the development and/or maintenance of a social network. Developing skills to establish an open dialogue and preserving friendships may help reduce anxiety when reintegrating into social roles and help maintain recovery when discharged. Patients may benefit from utilising friendship maps, a brief group intervention technique that helps patients hold friends in mind and prompts them to maintain contact [72]. Whilst some patients noted the artificial nature of social skills training, many were keen to receive interventions focused on social problem-solving and interpersonal effectiveness skills, particularly around ways to fit in, make conversation and self-acceptance. Opportunities to meet new people and practice social skills in an inpatient unit are rare and therefore may need to be sought from outside of the service, for example via encouraging hobbies, participation in youth groups and pen pals.

Alternatively evidence-based structured interventions such as Cognitive Remediation and Emotion Skills Training (CREST), developed for adults with EDs which aims to address difficulties with social cognition and functioning and has shown promising results, [73] may also be acceptable for a younger cohort if appropriately adapted. Adolescent social skills training packages may also be useful (e.g. skills training for adolescents [74], Social Competence Training [75]). Peers and parents are often unaware of the most beneficial ways to provide support [76] and psychoeducation and support could be provided by services to help to re-establish connections and avoid the isolation experienced during inpatient treatment.

Consistent with clinical governance guidelines advocating user involvement in the planning and provision of care, this qualitative study helped identify difficulties in life domains in order to inform service and intervention development. By involving patients, presenting feedback on current provisions and informing clinical staff of patient’s experiences regarding social skills and functioning this research has added new knowledge, depth and richness to clinical provisions in ED and the existing literature.

Several limitations of this study warrant caution in interpretation of findings. The study was conducted with adolescent inpatients with the most severe form of illness which may limit external validity. Future research may wish to address this in different ED populations, including outpatients and community samples to further determine whether the reported difficulties constitute therapeutic targets. The current sample contained adolescents with a diagnosis of AN-restrictive subtype and therefore it remains unclear how social experiences and functioning vary as a function of ED subtype in adolescents, and future research may wish to address this. Male patients were poorly represented (n = 1), limiting the generalisability of the results. This was primarily due to the hospital’s capacity regarding the number of male patients admitted at any one time. Although a disorder reported to be more prevalent in females, given the increasing prevalence in males, future research may wish to evaluate possible gender differences in social skills and functioning. Patients were also at different stages of treatment, potentially affecting their responses. In this context, the addition of social functioning and quality of life outcome measures (e.g. Work and Social Adjustment Scale [7]) or Clinical Impairment Assessment [77] may have been beneficial to examine and monitor progress and how much patients perceived that their ED impacted their everyday functioning. Future research may wish to integrate these quantitative and qualitative methodologies.

In conclusion, adolescent inpatients with EDs reported significant difficulties with social functioning and skills which appeared to be over and above the social difficulties typically reported at this stage of the lifespan. Future work may wish to develop and measure the impact of interventions such as CREST in younger cohorts to support these difficulties and enhance the likelihood of a successful recovery.

### Reflective Summary (KP)

I found working with this clinical population both challenging and rewarding. Facing silence in the first two focus groups we conducted was surprising and difficult to overcome and made me question the appropriateness of the semi-structured guide. Even though I knew this was a potentially challenging topic I believed the patients would be open to discussing and sharing experiences with their peers, and even though this eventually happened it took longer than anticipated. I was also surprised by the apparent change in demeanour and behaviour in some patients, during the focus group (where they would be shy and not maintain eye contact) and straight after (where they became loud and animated). This made me think there was a strong influence of group dynamics and perceptions within these young people, which I had to bear in mind during subsequent data collection and analysis. What pleasantly surprised me was how enthusiastic patients were to hear the research outcomes even though they were initially sceptical about taking part.
